# Spatial omics and AI for clinically actionable cancer biomarkers

**DOI:** 10.1371/journal.pmed.1005049

**Published:** 2026-04-09

**Authors:** Nic G. Reitsam

**Affiliations:** 1 Pathology, Faculty of Medicine, University of Augsburg, Augsburg, Germany; 2 Else Kroener Fresenius Center for Digital Health, Technical University Dresden, Dresden, Germany; 3 Bavarian Cancer Research Center (BZKF), Augsburg, Germany

## Abstract

In this Editorial, Nic Reitsam discusses how integrating spatial omics with artificial intelligence is likely to advance biomarker research and diagnostics, including the potentials and challenges of developing scalable, reproducible quantification in routine cancer pathology.

## Teaser

Integrating spatial omics with artificial intelligence is likely to advance biomarker research and diagnostics, with the potential to pair mechanistic insight into spatial target biology. By developing scalable, reproducible quantification in routine pathology, it can help bridge discovery, validation, and real-world clinical implementation.

Cancer care increasingly hinges on a deceptively simple question: Is a tumor “positive” or “negative” for a given biomarker? These labels determine eligibility for a fast-growing set of targeted treatments against antigens, such as HER2, TROP2, NECTIN4, and CLDN18.2. These therapies share a basic premise: find a molecular target and use it to deliver a lethal hit. Antibody-drug conjugates (ADCs) ferry cytotoxic payloads into antigen-expressing cells; immune and cellular approaches aim to focus immune recognition on those targets; and radiopharmaceutical therapies use target-binding molecules to deliver radiation directly to tumor tissue. The promise is precision: greater effect where the target is, less where it is not. But that promise rests on an assumption that often fails in reality: that target expression is uniform, when in fact it is heterogeneous within lesions, across space, and over time. Therapeutic efficacy depends not only on whether a target is present, but on where it is expressed, at what level and within which microenvironmental context, turning “target-positive” into a spatial, quantitative measurement problem.

## Spatial single-cell omics makes target biology interpretable and testable

Spatial profiling reframes tissue as structured ecosystems rather than homogeneous samples [1]. Spatial transcriptomics at single-cell resolution and multiplex spatial proteomics allow interrogation of where target-expressing cells reside, how they interact with their microenvironment, and whether target-negative reservoirs coexist that may seed resistance or relapse.

Recent spatial atlases of adult and pediatric brain tumors illustrate this challenge clearly. Antigens such as B7-H3, EGFR, or IL13RA2 may be restricted only to a subpopulation of cells within a tumor, while recurrence coincides with reorganization of tumor-immune niches [2,3], where interactions between cancer, immune, and stromal cells can either support immune attack or, conversely, enforce immune evasion. Importantly, biologically critical regions are often underrepresented in bulk or average-based assays despite their disproportionate role in invasion, dissemination, and metastatic seeding [4], challenging simplistic assumptions that average expression captures therapeutic vulnerability.

## Overcoming bottlenecks with computational pathology and AI

A central challenge is not only generating spatial data, but also making it comparable over time, sites, and platforms, as well as reproducible and scalable. This is where computational pathology and artificial intelligence (AI) become indispensable.

In practice, reproducibility is substantially influenced by pre-analytics (e.g., fixation, staining, scanner variation, sampling) and inter-observer variability. To address this, target-specific computational pathology biomarkers have begun to emerge. For example, quantitative scoring of the biomarker PD-L1 from whole-slide images aims to replace subjective categories by estimating continuous measures (e.g., percent positive cells, intensity distributions) at scale [5,6]. Similar quantitative computational approaches are emerging for ADC targets such as TROP2, where heterogeneous, predominantly membrane-localized staining makes coarse cutoffs particularly fragile [7]. In parallel, AI models are increasingly explored to infer higher-dimensional spatial information directly from routine histopathology (commonly Hematoxylin and Eosin (H&E) stainings). “Virtual” transcriptomic/proteomic methods learn associations between tissue morphology and the underlying transcriptomic/proteomic phenotype from paired training data, enabling the prediction of virtual spatial immune maps from standard slides [8,9]. Recently, an approach combining H&E morphology with virtual spatial proteomics to predict immunotherapy response in lung cancer outperformed clinically established biomarkers such as PD-L1 expression and tumor mutational burden [8]. However, such results require critical validation. Importantly, correlations between virtual predictions and actual protein expression are frequently moderate to weak depending on the marker, indicating that morphology-based approaches likely have inherent ceilings, particularly for markers lacking visible morphological correlates. Nevertheless, such predictions still may correlate with clinically meaningful endpoints such as treatment response or survival. This raises a difficult interpretive question: if a virtual assay predicts clinical benefit without accurately reflecting true target expression, should such predictions be integrated into diagnostic workflows and treatment decisions? Meanwhile, foundation models for multiplex imaging (large AI models pretrained on diverse datasets that can be adapted to a wide range of downstream tasks, including cell-type classification and biomarker discovery) [10] aim to harmonize representations across markers and platforms by learning generalizable features. Although they are unlikely to translate directly into clinical use in the very near future, they may advance large-scale biomarker discovery.

## From threshold testing to longitudinal decision support

A clinical example where therapeutic advances can rapidly outpace existing biomarker frameworks and complicate routine biomarker reporting is in HER2 grading. Trials of the ADC trastuzumab deruxtecan (T-DXd) have introduced the distinction between HER2-low, HER2-ultralow, and HER2-zero. Here, the phase III DESTINY-Breast06 trial of T-DXd reports improved progression-free survival versus chemotherapy in metastatic breast cancer patients with hormone receptor-positive, HER2-low metastatic breast cancer, while analyses in HER2-ultralow disease were exploratory but directionally consistent [11]. This forces routine distinction of HER2-zero from HER2-ultralow, where assay variability and reproducibility become limiting, although the clinical relevance of this distinction remains under ongoing debate [12]. This redefinition shifts HER2 assessment from categorical scoring to a borderline quantification problem, where inter-observer variability is highest; accordingly, AI-based immunohistochemistry analysis has demonstrated very high sensitivity for identifying T-DXd-eligible tumors (pooled sensitivity ~97%, corresponding to only ~3% missed eligible cases) [13], while human assessment shows substantially lower agreement at the critical low-expression thresholds (≤70% agreement [14]), supporting its role in improving reproducibility at these decision boundaries [13].

This highlights the need for quantitative, standardized reporting and, where necessary and possible, new assays and biomarkers aligned to therapeutic mechanisms rather than historical thresholds. Practically, it also argues for trial designs that treat biomarker positivity as a continuous, spatial phenotype rather than a one-time binary label. Consider a concrete example: instead of a pathology report stating “HER2-low,” in the future a clinician might receive a report that only 15% of tumor cells show weak but detectable expression, clustered in a stromal-rich, immune-excluded tumor region, raising the question of whether such subclonal, low-level positivity in a microenvironment with limited immune cell and drug access is sufficient to drive meaningful therapeutic response.

Three shifts follow naturally. First, trials should use continuous measures, such as percentage of expressing cells and staining intensity, to stratify patients, rather than collapsing this information into binary cutoffs. Second, biomarker assessment should not end at baseline: repeating it at progression can reveal whether an initially target-positive tumor has lost expression. Third, trial platforms should be adaptive enough to update a patient’s stratification as new biopsy or imaging data come in, rather than a single pretreatment snapshot.

In routine practice, the near-term path could be a hybrid workflow. On the one hand, we should strengthen what can be deployed widely: robust, low-cost assays built on routine pathology, supported by computational pathology to deliver reproducible and quantitative readouts, including discovery of deployable H&E biomarkers when deep learning aligns with human-recognizable phenotypes. On the other hand, spatial single-cell omics and other high-dimensional assays remain essential to explain biological mechanisms, map heterogeneity, and define the next generation of biomarkers, but should not yet be assumed to be scalable for clinical use.

## Precision that scales

The challenge is to advance spatial omics and AI biomarkers and the infrastructure they require without losing sight of clinical reality and widening disparities. High-cost platforms (often exceeding $250,000 per instrument) require dedicated bioinformatics expertise, substantial data storage (with several gigabytes per tissue slide), and trained personnel; these resources will remain inaccessible in many settings, particularly in low- and middle-income countries. Hence, clinically actionable biomarkers should build on widely deployable assays, complemented by selective spatial or AI-based assays in trials and specialized centers to calibrate biomarkers, track tumor evolution, and prepare for broader adoption as these methods mature.

Achieving this will also require policy updates that address the practical barriers to scale and clinical adoption. For example: Reimbursement pathways for AI-based and spatial omics assays, if those assays prove clinically beneficial; repeat testing frameworks that recognize biomarker evolution; multicenter ring trials with proficiency testing to ensure cross-site reproducibility; transparent, versioned reporting of data, models, and updates to support regulatory oversight and clinical trust; equity guardrails and access benchmarks to prevent widening disparities; and structured training for clinicians, pathologists, and trial teams to enable safe and consistent use.

Ultimately, the goal is not technology for its own sake but a diagnostic ecosystem in which highly resolved spatial biology informs what to measure, AI determines how to measure it, and both are embedded in workflows that improve outcomes across settings. Getting there will require the same rigor we demand of every other new biomarker or drug: prospective validation, honest reporting of what works and what doesn’t, and a commitment to making advances accessible beyond the institutions that develop them.

## Abbreviations

ADCs: antibody-drug conjugates
AI: artificial intelligence
H&E: Hematoxylin and Eosin
T-DXd: trastuzumab deruxtecan
